# Collembolan species diversity of calcareous canyons in the Republic of Moldova

**DOI:** 10.3897/zookeys.506.8643

**Published:** 2015-06-01

**Authors:** Galina Buşmachiu, Anne Bedos, Louis Deharveng

**Affiliations:** 1 Institute of Zoology of Academy of Sciences of Moldova, Academiei str.1, 2028 Chişinău, Moldova; 2 Institut de Systématique, Evolution, Biodiversité, ISYEB - UMR 7205 - CNRS, MNHN, UPMC, EPHE, Museum national d'Histoire naturelle, Sorbonne Universités, 45 rue Buffon (CP50), 75005 Paris, France

**Keywords:** Biodiversity, flotation method, checklist, life form

## Abstract

The study of collembolan communities from the Vîşcăuți canyon in Moldova revealed 63 species belonging to 41 genera and 12 families, including four species new for the fauna of the Republic of Moldova. A checklist of collembolan species identified in the five calcareous canyons sampled so far in Moldova is included, with data on habitats, life form, occurrence and comments of distribution of most remarkable species. Of the 98 recognized species of these calcareous canyons, only 38 were shared by Vîşcăuți and the other canyons. The richness of calcareous habitats together with the high heterogeneity in faunal composition suggests that further significant increase in the species richness of the region may be expected.

## Introduction

Republic of Moldova has a rather small territory (33,760 km^2^) but its heterogeneous natural conditions and its geographical position contributed to the formation of diverse types of soils, supporting high diversity of flora and fauna.

The largest river in the country is the Dniester. Its length within the territory of Moldova is 657 km, its catchment representing about 70% of the territory of country. The Dniester riverbed is sinuous in its upper course penetrating calcareous formations that emerge to the ground surface as cliffs and rocky banks. Along the course of water, petrophyte ecosystems are common and occupy a surface of 23 000 ha, being formed on the submarine coral reefs of Sarmatian Sea, emerged more than 10 million years ago.

The petrophyte ecosystems in calcareous canyons of Dniester River are characteristic elements of the landscape – unique in the north-western part of Black Sea basin.

The first result of the study concerning collembolan fauna of these canyons reported 56 species collected from five localities (Buşmachiu 2011a). The survey of Collembolan diversity on all riparian habitats of the Dniester revealed 138 species (Buşmachiu 2011b); however, it involved habitats such as natural steppe or natural flooded and xerothermic forests, which were not represented in Vîscăuți, the canyon object of the present study. Only 14 species were reported from Vîscăuți in our last paper.

The present study was part of collembolan fauna survey carried out in the calcareous canyons of the Dniester River and allowed us to identify one genus (*Appendisotoma*) and four species new for the fauna of the Republic of Moldova.

## Material and methods

### Study sites

**Figure 1.**
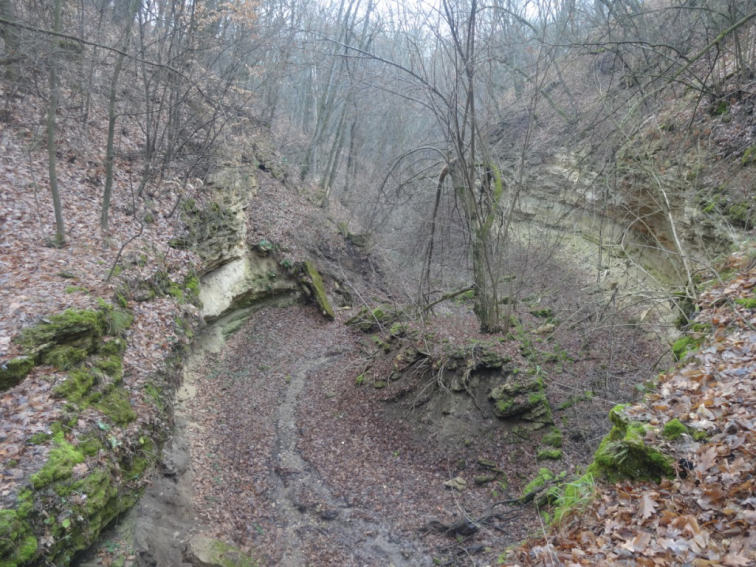
Calcareous canyon on the bank of Dniester River near the locality of Vîșcăuți.

The samples were taken in a calcareous canyon near the locality of Vîşcăuți situated close to the Dniester River in the central part of Moldova (47°43'N, 29°07'E, altitude 52 m). Canyon slopes are steep and covered with natural deciduous forest (Fig. 1). The trees trunks growing on limestone slopes and calcareous rocks are covered with moss and lichens. After each rain, water is drained from the surroundings into the canyon, where soils at the bottom are usually very wet and covered with moss.

Several types of habitats and microhabitats of the canyon were sampled for the study (Table 1). The samples were collected randomly in November 2009 (8 samples), May 2010 (4 samples) and January 2014 (13 samples), amounting to a total number of 25. Litter and soil were sampled by a metallic square frame of 25 cm^2^ for 5 cm depth, each sample including 4 subsamples. Decaying wood, moss and lichens were taken additionally by hand. The winter 2013-2014 was very warm in Moldova and the first frosts began after our sampling within January, 2014, that could partly explain the richness of the collected fauna.

**Table 1.** The types of studied habitats and number of samples from the Vîşcăuți canyon.

Studied habitats	Litter + soil	Soil	Litter	Lichen on wood	Moss on soil	Moss on rock	Barks of trees	Decaying wood	Number of samples
	LS	S	L	LW	MS	MR	B	DW	
Bottom of the canyon	3	1		1	1	1			7
Forest on slopes	3	1				2	1	3	10
Trees above the canyon	2		3			1	1		7
Pasture		1							1
Number of samples	8	3	3	1	1	4	2	3	25

### Extraction method and identification

**Figure 2.**
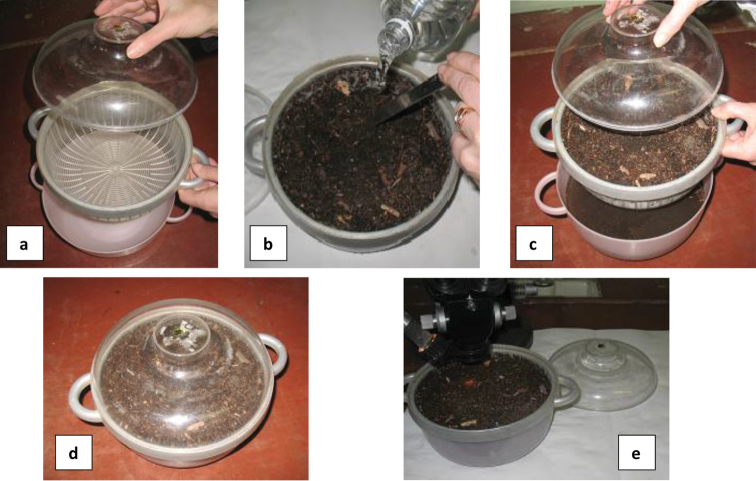
Extraction of Collembola using flotation method (**a** plastic containers **b** filling the containers with sample and water **c** covering the containers **d** soaking the sample **e** collecting floating specimens under stereomicroscope).

The microarthropods were extracted from the soil using a modified flotation method (Fig. 2). A round plastic container of 1.8 litters and 22 cm in diameter was used for extraction. The container is compound of two elements with handles and a cap, the internal one being perforated by many holes (Fig. 2a). The sample to be extracted is put into the internal container and water is added in the containers (Fig. 2b) and they are covered with a cap (Fig. 2c). The soaking of the sample takes no more than 5–15 minutes depending on the soil structure (Fig. 2d) for getting Collembola out of the substrate. Then container is shaken slowly several times and the sample is stirred with a spatula. This process done carefully allows the collembolan specimens to move up to the surface of the water. Neanuridae and Tullbergiidae need more time than others to break surface. The floating collembolan specimens are collected one by one by hand under binocular (Fig. 2e), using entomological needle or disposable syringe with the end of the needle curved.

Specimens were stored in 96% ethyl alcohol and counted. They were cleared in lactic acid and KOH and mounted on slides using Marc Andre II medium. Identification was mainly done with a phase contrast microscope LEICA 2500 equipped with camera Lucida, using the standard determination keys and recently published Synopses on Palaearctic Collembola (Bretfeld 1999; Potapov 2001; Thibaud et al. 2004; Dunger and Schlitt 2011; Jordana 2012).

## Results and discussion

As a result of our survey, 63 species of Collembola belonging to 41 genera and 12 families were found in the Vîşcăuți canyon. The family Entomobryidae was represented by 14 species, followed by the families Isotomidae – 13, Tullbergiidae – 9, Neanuridae – 8, Hypogastruridae – 6, Onychiuridae – 4, Neelidae and Odontellidae – 2 species, Tomoceridae, Arrhopalitidae, Katiannidae, Dicyrtomidae and Sminthurididae with one species each (Table 2). One genus – *Appendisotoma* Stach, 1947 and four species – *Jevania fageticola* Rusek, 1978, *Appendisotoma abiskoensis* (Ǻgrell, 1939), *Appendisotoma absoloni* Rusek, 1966 and *Folsomia volgensis* Martynova, 1967 are new for the Republic of Moldova.

**Table 2.** Collembolan species found in the studied canyons. * – species new for the fauna of the Republic of Moldova; OC – other studied canyons; O – biogeographic occurrence (C – cosmopolitan, E – European, H – Holarctic, P – Palaearctic, M – Mediterranean, R – endemic); LF – life forms (e – epiedaphic, h – hemiedaphic, eu – euedaphic); abbreviations for habitats are given in Table 1.

Taxon	Number of specimens		Habitats	LF	O
	Vîscăuți	OC			
**Hypogastruridae**					
*Ceratophysella engadinensis* (Gisin, 1949)	2 ex.	7 ex.	L	e	C
*Ceratophysella* sp. juv.	1 ex.		DW	e	-
*Hypogastrura manubrialis* (Tullberg, 1869)	2 ex.		L	e	C
*Schoettella ununguiculata* (Tullberg, 1869)		5 ex.	L	e	H
*Xenylla boerneri* (Axelson, 1905)	24 ex.		B, MR, DW	h	E
*Xenylla brevisimilis brevisimilis* Stach, 1949		23 ex.	L, LS, LW	h	E
*Xenylla corticalis* Börner, 1901	19 ex.		MS, DW	h	E
*Xenylla maritima* Tullberg, 1869	8 ex.	10 ex.	L, LS	h	C
*Xenylla uniseta* Gama, 1963		12 ex.	MR	h	M
**Neanuridae**					
*Friesea mirabilis* (Tullberg, 1871)		7 ex.	L	h	C
*Deutonura albella* (Stach, 1920)	1 ex.	5 ex.	DW	h	E
*Deutonura stachi* (Gisin, 1952)		4 ex.	L	h	E
*Endonura gracilirostris* Smolis, Skarżyński, Pomorski & Kaprus’, 2007	2 ex.	1 ex.	DW	h	E
*Lathriopyga nistru* Buşmachiu, Deharveng & Weiner, 2010	3 ex.	10 ex.	L, DW	h	R
*Neanura moldavica* Buşmachiu & Deharveng, 2008	11 ex.	15 ex.	L, DW	h	R
*Neanura minuta* Gisin, 1963		1 ex.	DW	h	E
*Neanura muscorum* (Templeton, 1835)	2 ex.		L, DW	h	C
*Micranurida pygmaea* Börner, 1901	7 ex.	4 ex.	L, MS, MR, DW	eu	C
*Pseudachorutes parvulus* Börner, 1903	35 ex.		L	e	E
*Pseudachorutes pratensis* Rusek, 1973		1 ex.	L	e	E
*Pseudachorutes subcrassus* Tullberg, 1871	5 ex.	6 ex.	L, MR, DW	e	P
**Odontellidae**					
*Axenyllodes bayeri* Kseneman, 1935	3 ex.		S	eu	E
*Superodontella montemaceli* Arbea & Weiner, 1992	1 ex.	1 ex.	L	h	E
**Onychiuridae**					
*Dimorphaphorura irinae* (Thibaud & Taraschuk, 1997)	3 ex.		S	eu	E
*Kalaphorura paradoxa* (Schäffer, 1900)		~ 47 ex.	L, S	eu	E
*Micraphorura uralica* (Khanislamova, 1986)	13 ex.	25 ex.	L, S	eu	P
*Protaphorura armata* (Tullberg, 1869)		7 ex.	S	eu	C
*Protaphorura pannonica* (Haybach, 1960)		3 ex.	S	eu	E
*Protaphorura sakatoi* (Yosii, 1966)	79 ex.	~ 37 ex.	S, L, MS	eu	E
*Protaphorura subarmata* (Gisin, 1957)	103 ex.	~ 59 ex.	S, L, MS	eu	E
*Thalassaphorura tovtrensis* (Kaprus’ & Weiner, 1994)		81 ex.	L, S	eu	E
**Tullbergiidae**					
*Doutnacia xerophila* Rusek, 1974	4 ex.	6 ex.	S	eu	E
*Jevania fageticola Rusek, 1978	3 ex.		S	eu	E
*Jevania weinerae* Rusek, 1978		3 ex.	L	eu	E
*Karlstejnia rusekiana* Weiner, 1983	1 ex.		S	eu	E
*Mesaphorura critica* Ellis, 1976	21 ex.	5 ex.	S, LW	eu	P
*Mesaphorura florae* Simón, Ruiz, Martin & Luciáňez, 1994	6 ex.		S	eu	E
*Mesaphorura jarmilae* Rusek, 1982	1 ex.		S	eu	E
*Mesaphorura hylophila* Rusek, 1982	4 ex.	9 ex.	S	eu	P
*Mesaphorura italica* (Rusek, 1971)		2 ex.	S	eu	P
*Mesaphorura krausbaueri* Börner, 1901		7 ex.	S	eu	P
*Mesaphorura macrochaeta* Rusek, 1976	1 ex.		S	eu	C
*Mesaphorura sylvatica* (Rusek, 1971)		2 ex.	S	eu	P
*Mesaphorura yosii* (Rusek, 1967)		6 ex.	S	eu	C
*Metaphorura affinis* (Börner, 1902)	1 ex.	2 ex.	S	eu	P
**Isotomidae**					
*Appendisotoma abiskoensis (Ågrell, 1939)	24 ex.		L	e	E
*Appendisotoma absoloni Rusek, 1966 juv.	15 ex.		L	e	E
*Folsomia quadrioculata* (Tullberg, 1871)	11 ex.	~ 26 ex.	L	h	H
*Folsomia manolachei* Bagnall, 1939	7 ex.	7 ex.	L	h	P
*Folsomia penicula* Bagnall, 1939		11 ex.	L, MR	h	P
*Folsomia volgensis Martynova, 1967	27 ex.		L	h	P
*Folsomides angularis* (Axelson, 1905)		7 ex.	LS	h	H
*Folsomides marchicus* (Frenzel, 1941)		37 ex.	LS	h	E
*Folsomides parvulus* Stach, 1922	3 ex.	~75 ex.	LS	h	C
*Desoria olivacea* (Tullberg, 1871)	1 ex.		L	e	H
*Isotoma riparia* (Nicolet, 1842)	1 ex.		B	e	E
*Isotoma viridis* Bourlet, 1839	14 ex.	18 ex.	L, MR	e	H
*Isotomiella minor* (Schäffer, 1896)	9 ex.	13 ex.	L, MS, DW	eu	H
*Isotomodes productus* (Axelson, 1906)	1 ex.	12 ex.	LS	eu	C
*Parisotoma notabilis* (Schäffer, 1896)	58 ex.	~124 ex.	LS, MR, MS, DW	h	C
*Proisotomodes bipunctatus* (Axelson, 1903)		~ 23 ex.	DW	h	E
*Vertagopus* sp.	2 ex.		L	e	-
**Entomobryidae**					
*Heteromurus major* (Moniez, 1889)	5 ex.	~ 15 ex.	L	e	M
*Heteromurus nitidus* (Templeton, 1835)		3 ex.	MR	e	C
*Entomobrya nigrocincta* Denis, 1923		2 ex.	L	e	E
*Entomobrya marginata* Tullberg, 1871	21 ex.		MS, L, B	e	E
*Entomobrya multifasciata* (Tullberg, 1871)	2 ex.	5 ex.	M	e	H
*Entomobrya nivalis* (Linnaeus, 1758)	2 ex.		Moss	e	C
*Lepidocyrtus curvicollis* Bourlet, 1839	1 ex.		L	e	H
Lepidocyrtus gr. lignorum (Fabricius, 1775)	56 ex.	~ 38 ex.	L	e	H
*Lepidocyrtus paradoxus* Uzel, 1890		4 ex.	L, MR	e	H
*Lepidocyrtus violaceus* Lubbock, 1873		7 ex.	L	e	H
*Orchesella cincta* (Linnaeus, 1758)		1 ex.	L	e	H
*Orchesella maculosa* Ionesco, 1915	7 ex.	3 ex.	MR	e	E
*Orchesella multifasciata* Stscherbakow, 1898	2 ex.	4 ex.	L, MR	e	E
*Orchesella orientalis* Stach, 1960		2 ex.	MR	e	E
*Orchesella pseudobifasciata* Stach, 1960	37 ex.	4 ex.	M, L	e	E
*Orchesella xerothermica* Stach, 1960	4 ex.		L, MR	e	E
*Pseudosinella horaki* Rusek, 1985	24 ex.	~ 32 ex.	L, MS, DW	h	E
*Pseudosinella imparipunctata* Gisin, 1953	1 ex.	11 ex.	L	h	E
*Pseudosinella moldavica* Gama & Buşmachiu, 2002	3 ex.	28 ex.	L	h	E
*Pseudosinella octopunctata* Börner, 1901		18 ex.	L	h	C
*Seira domestica* (Nicolet, 1842)	1 ex.	1 ex.	L	e	E
**Tomoceridae**					
*Pogonognathellus flavescens* (Tullberg, 1871)		5 ex.	DW	h	H
*Tomocerus minor* (Lubbock, 1862)		2 ex.	L	h	C
*Tomocerus vulgaris* (Tullberg, 1871)	2 ex.		DW	h	C
**Cyphoderidae**					
*Cyphoderus albinus* Nicolet, 1842		3 ex.	L	eu	P
*Cyphoderus bidenticulatus* Parona, 1888		7 ex.	S, L	eu	M
**Neelidae**					
*Megalothorax minimus* Willem, 1900	8 ex.	12 ex.	L, S	eu	C
*Neelus murinus* Folsom, 1896	6 ex.	6 ex.	L, S	eu	C
**Sminthurididae**					
*Sphaeridia pumilis* (Krausbauer, 1898)	1 ex.	~21 ex.	L, MR	h	C
**Arrhopalitidae**					
*Pygmarrhopalites* sp.	3 ex.		DW	eu	-
**Katiannidae**					
*Sminthurinus aureus* (Lubbock, 1862)	7 ex.	2 ex.	L	e	P
*Sminthurinus elegans* (Fitch, 1863)		4 ex.	L	e	E
*Sminthurinus niger* (Lubbock, 1868)		5 ex.	L	e	P
**Sminthuridae**					
*Caprainea marginata* (Schott, 1893)		2 ex.	L	e	P
**Dicyrtomidae**					
*Dicyrtoma minuta* (Fabricius, 1763)	1 ex.	1 ex.	L, MR	e	E
*Ptenothrix leucostrigata* Stach, 1957		2 ex.	L	e	E
**Total number of species: 98**	**63**	**73**			

The first study concerning collembolan fauna of calcareous canyons near the localities Lalova, Țipova, Saharna, Vîşcăuți and Butuceni with similar ecological settings recorded 56 species (Buşmachiu 2011a). One additional species was included in the next paper (Buşmachiu 2011b).

The present research increases the number of Collembola species revealed from the calcareous canyons of the Republic of Moldova from 57 to 98, which belong to 49 genera and 15 families. In Vîşcăuți were recorded 63 species, in other four localities 73. Only 38 species were shared by Vîşcăuți and the other canyons. Because none of the species of the canyons are considered local micro-endemics, this high divergence in faunal composition may result from important differences in sampled habitats.

The comparison with canyons of Lalova, Țipova, Saharna and Butuceni (below named as “other canyons” – OC) revealed that contribution of the different Collembolan families to local biodiversity was similar, with the dominance of two families (Table 2): Entomobryidae with 21 species (14 species in Vîşcăuți and 17 in OC) and Isotomidae with 17 species (13 and 11). They are followed by the families Tullbergiidae with 14 species (9 and 9), Neanuridae with 12 species (8 and 10), Hypogastruridae with 9 species (6 and 5) and Onychiuridae with 8 species (4 and 7). Two families comprised three species: Tomoceridae (1 and 2) and Katiannidae (1 and 3); four families – two species: Odontellidae (2 and 1), Neelidae (2 and 2), Dicyrtomidae (1 and 2) and Cyphoderidae (0 and 2); other three families, Sminthurididae, Sminthuridae and Arrhopalitidae were represented by one species each. The family Arrhopalitidae missed in the other canyons, while Cyphoderidae and Sminthuridae were not found in Vîşcăuți (Table 2).

The distribution and ecology of the most interesting and rare taxa through the country is commented below.

Among the Collembola collected in the canyons, the family Hypogastruridae includes 9 species and 4 genera. In the Republic of Moldova the genus *Xenylla* is represented by seven species (Buşmachiu and Weiner 2008). Five of them were present in studied canyons. Populations of this genus are usually represented by numerous specimens in moss, litter and sometimes on the bark of trees. Though all species are largely distributed in Europe, their distribution among studied canyons differs greatly, with only one species shared by Vîşcăuți and other canyons. This may point again to differences in sampled habitats.

The family Neanuridae was represented by 12 species from 7 genera. The most interesting and most diversified among them are Neanurinae, all linked to litter and decaying wood in Moldova, with two species probably endemic for the country (*Lathriopyga nistru* and *Neanura moldavica*), and one species originally described as endemic of Crimea (*Endonura gracilirostris*) (Buşmachiu and Deharveng 2008; Buşmachiu et al. 2010; Smolis et al. 2007). These three species are frequent in all or most of the canyons. All are typical species for calcareous soils situated along the Dniester River, but are also present in the natural forest and under lower shrubs throughout the country (Buşmachiu 2008). *Neanura minuta*, of much larger distribution in Europe, is a very rare species in Moldova, only found in low number in the litter of the Saharna canyon (Buşmachiu 2011b).

In the calcareous canyons the family Onychiuridae was represented by 8 species from 5 genera. The species typical for calcareous soil – *Kalaphorura paradoxa* was found in every canyon covered with natural forest or rare lower shrubs except Vîşcăuți. Pomorski (1998) cited it as living in humid litter of mountains, under stones and pieces of wood: this is a rather different ecology, and suggests that two forms may be included under this species name. *Thalassaphorura tovtrensis* was found along a streamlet in Butuceni, i.e. in same ecological conditions as its occurrence outside Moldova (Kaprus’ and Weiner 1994; Thibaud et al. 1999). The species *Micraphorura uralica* is widespread from Bashkiria in the south part of Ural Mountain (Khanislamova 1986) to Ukraine, and inhabits forest litter and moss on stone (Kaprus’ and Weiner 1994, Kaprus’ et al. 2002).

The smallest species of Poduromorpha belong to Tullbergiidae, which are well diversified in European soils. In Moldavian calcareous canyons, 14 species and 5 genera were collected. Among them, 5 species are only present in Vîşcăuți, while 5 are absent from this canyon, pointing once again to the originality of its faunal composition. Few species (4 out of 14) were present in Vîşcăuți and in OC. *Mesaphorura italica*, *Mesaphorura macrochaeta*, *Mesaphorura sylvatica* and *Mesaphorura yosii* were especially observed in open habitats of calcareous canyons covered with low shrubs, and are not present in Vîşcăuți. The species *Mesaphorura jarmilae* and *Karlstejnia rusekiana*, only cited previously from the soils of natural deciduous forest, are here recorded in Vîşcăuți. The genus *Jevania* includes only two rare silvicolous species in Europe. Both of them inhabit the soils in Moldova, with *Jevania weinerae* only cited from calcareous soil of Lalova canyon (Buşmachiu and Weiner 2010) and *Jevania fageticola* cited from Vîşcăuți (first record for Moldova). *Karlstejnia rusekiana* is a silvicolous euedaphic species described from a cave of southern Poland in beech and oak-hornbeam forest area (Weiner 1983) and cited from Ukrainian forest (Kaprus’ et al. 2006).

**Figures 3.**
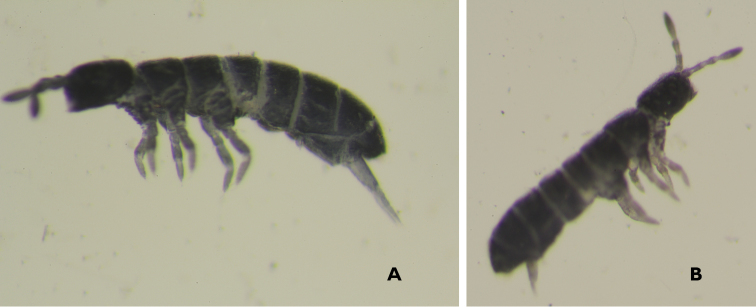
**A** *Appendisotoma abiskoensis* **B** *Appendisotoma absoloni*.

Family Isotomidae was represented in the canyons by 17 species from 10 genera. The most speciose genera were *Folsomia* and *Folsomides*. The most interesting, and new for Moldova, was the genus *Appendisotoma*. *Folsomides angularis* and *Folsomides marchicus* were present in some of the studied canyons, mostly in open habitat or under lower shrubs, but not found in Vîşcăuți; they have a similar ecology in Europe (Potapov 2001). Species from genus *Folsomia* may inhabit several types of ecosystems, including disturbed ones. Four species of the genus were found in Moldavian calcareous canyons, of which one, *Folsomia volgensis*, is cited for the first time in the country. This species inhabits forest – steppe region in central part of the Palaearctic region, being common in dry forest biotopes (Potapov 2001) and is very rare in the Ukrainian fauna (Kaprus’ et al. 2006). Two species of *Appendisotoma*, *Appendisotoma abiskoensis* (Fig. 3A) and *Appendisotoma absoloni* (Fig. 3B), were identified. Both are first records for Moldova, and so far restricted to Vîşcăuți canyon where they were collected in January, in litter. The first species is considered rare, recorded in litter and moss near the streams (Potapov 2001) and also cited from the Ukrainian steppe (Kaprus’ et al. 2006). *Appendisotoma absoloni* is rather common in Czech deciduous forest, mostly abundant in autumn (Rusek 1968). Remarkably, these two species were collected in a same sample, both in large number.

The total number of Entomobryidae represented in the studied canyons was 21 species from 6 genera. Among species living preferentially in moss on limestone in Moldova are several *Orchesella* and *Entomobrya* species. One of them, *Orchesella maculosa* was found in most studied canyons and not in other ecological conditions. This species was first cited from calcareous places near caves in south-western Romania (Ionesco 1915), and then from meadows near the Dniester canyon of the Ukrainian part of the river (Chernobai et al. 2003).

The microhabitats of the studied locality are rich in rare species from families Isotomidae (*Appendisotoma abiskoensis*, *Appendisotoma absoloni*, *Folsomia volgensis*), Tullbergiidae (*Karlstejnia rusekiana*, *Jevania fageticola*) and Onychiuridae (*Dimorphaphorura irinae*), but poor in Symphypleona species. Symphypleona species are rare not only in microhabitats of calcareous canyons, but also in the riparian habitats exposed to periodical flooding on the bank of Dniester River (Buşmachiu and Weiner 2013). The species of this group, especially from the families Dicyrtomidae, Katiannidae and Sminthuridae, are abundant in the herbaceous plants of open habitats (Buşmachiu 2011b). Their rarity (3 species, versus 7 for OC) in our samples may result from a lack of favourable open habitats in Vîşcăuți, but also by unadapted sampling techniques, as suggested by the abundance of Neelipleona and soil-dependent species of Symphypleona (*Sphaeridia pumilis*, *Sminthurinus aureus*, *Pygmarrhopalites* sp.).

The Dniester flows through Ukraine and Moldova. It is therefore not surprising that more than 90 collembolan species revealed in our study are shared with Ukraine. The analysis of collembolan species from studied calcareous canyons brings new information on the peculiarities of species distribution and on their ecological preferences. Of special interest is the fact that, for several families, a large proportion of the species are not shared by Vîşcăuți and other canyons. This may be due to differences more important than estimated at first sight that may exist in sampled habitats.

The three classical morpho-functional groups of epiedaphic, hemiedaphic and euedaphic were represented by a quite similar number of species, i.e. 35 epiedaphic, 31 hemiedaphic, and 32 euedaphic (Table 2). These groups differ in dispersal ability and other life traits such as reproduction, mobility, metabolic activity and feeding behaviour (Hopkin 1997). In our dataset, they usually match the vertical gradient from surface to deep soil. So the petrophyte ecosystems covered by natural forest, with moss and decayed wood, provide diversified micro-microhabitats to a large diversity in each of these three functional groups.

**Figure 4.**
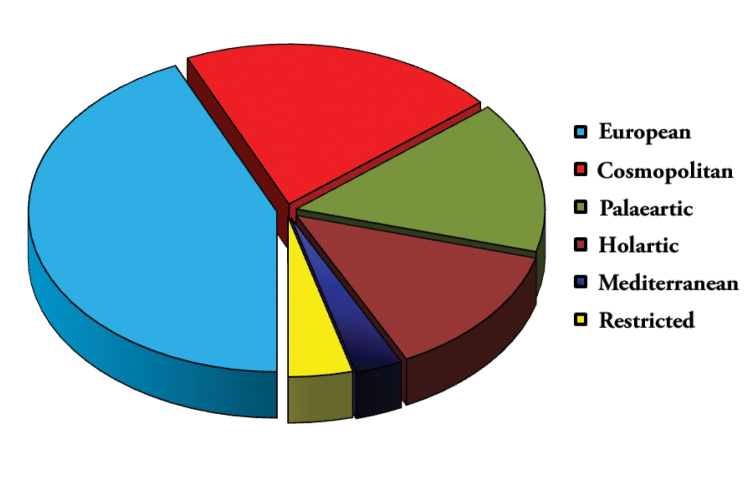
Percentage of identified collembolan species per biogeographical categories in the studied calcareous canyons.

The most part of identified species in the calcareous canyons have a wide occurrence (Fig. 4). Between them 43.3% have European, 20.7% – cosmopolitan, 15.3% – Palaearctic and 13.4% – Holarctic distribution. Only three species have Mediterranean distribution and two species were described from the Republic of Moldova; for the description of two other species, supplementary material is needed.

## Conclusions

With a total of 98 species of Collembola in 49 genera and 15 families, the fauna of the calcareous canyons of Moldova can be qualified of rich, though comparative data are lacking in other areas. Enlarging the spectrum of sampled habitats and collecting techniques (pitfall, berlesing, beating vegetation) will probably increase significantly this number, especially for Symphypleona. A second important result is the large differences in the composition of fauna between canyons. It is suggested that the relative importance of open versus forest habitats may explain most of these differences. At least, the presence of rare and even of a few endemic species may be noticed, giving a further interest to this Collembolan fauna of calcareous habitats. Additional sampling is currently carried out to check whether the originality of Vîşcăuți is real or an effect of sampling bias.
